# Dynamic genome-wide association analysis and identification of candidate genes involved in anaerobic germination tolerance in rice

**DOI:** 10.1186/s12284-020-00444-x

**Published:** 2021-01-06

**Authors:** Ling Su, Jing Yang, Dandan Li, Ziai Peng, Aoyun Xia, Meng Yang, Lixin Luo, Cuihong Huang, Jiafeng Wang, Hui Wang, Zhiqiang Chen, Tao Guo

**Affiliations:** National Engineering Research Center of Plant Space Breeding, South China Agricultural University, Guangzhou, 510642 China

**Keywords:** Rice, Anaerobic germination tolerance, Coleoptile, Dynamic GWAS, RNA-seq, Candidate gene

## Abstract

**Background:**

In Asian rice production, an increasing number of countries now choose the direct seeding mode because of rising costs, labour shortages and water shortages. The ability of rice seeds to undergo anaerobic germination (AG) plays an important role in the success of direct seeding.

**Results:**

In this study, we used 2,123,725 single nucleotide polymorphism (SNP) markers based on resequencing to conduct a dynamic genome-wide association study (GWAS) of coleoptile length (CL) and coleoptile diameter (CD) in 209 natural rice populations. A total of 26 SNP loci were detected in these two phenotypes, of which 5 overlapped with previously reported loci (S1_ 39674301, S6_ 20797781, S7_ 18722403, S8_ 9946213, S11_ 19165397), and two sites were detected repeatedly at different time points (S3_ 24689629 and S5_ 27918754). We suggest that these 7 loci (−log_10_ (*P*) value > 7.3271) are the key sites that affect AG tolerance. To screen the candidate genes more effectively, we sequenced the transcriptome of the flooding-tolerant variety R151 in six key stages, including anaerobic (AN) and the oxygen conversion point (AN-A), and obtained high-quality differential expression profiles. Four reliable candidate genes were identified: *Os01g0911700* (*OsVP1*), *Os05g0560900* (*OsGA2ox8*), *Os05g0562200* (*OsDi19–1*) and *Os06g0548200*. Then qRT-PCR and LC-MS/ MS targeting metabolite detection technology were used to further verify that the up-regulated expression of these four candidate genes was closely related to AG.

**Conclusion:**

The four novel candidate genes were associated with gibberellin (GA) and abscisic acid (ABA) regulation and cell wall metabolism under oxygen-deficiency conditions and promoted coleoptile elongation while avoiding adverse effects, allowing the coleoptile to obtain oxygen, escape the low-oxygen environment and germinate rapidly. The results of this study improve our understanding of the genetic basis of AG in rice seeds, which is conducive to the selection of flooding-tolerant varieties suitable for direct seeding.

**Supplementary Information:**

The online version contains supplementary material available at 10.1186/s12284-020-00444-x.

## Background

Cultivated rice (*Oryza sativa* L.) is a staple food for over half of the world’s population (Zhang et al. 2019). The world’s rice demand is projected to increase by 25% from 2001 to 2025 with a targeted production of 732.5 MT, which is achievable by the addition of 5.9 MT every year. However, based on population growth, the Food and Agricultural Organization estimates that by 2050, the world rice requirement will be 524 MT with an annual increase of 2 MT from the present level of production (Jeon et al. 2011).

Worldwide, irrigated rice ecosystems comprise 55% of the world’s rice-growing area and provide 75% of global rice production (Mahender et al. 2015). However, the current evidence shows a decline in grain yield productivity due to looming threats to natural resources, falling water tables, mounting labour shortages, energy scarcity, increasing input prices and changing climatic conditions (Singh et al. 2013). However, the water-, energy-, and labour-intensive system of transplanted puddled rice is steadily being replaced by direct-seeded rice (DSR) due to the progressive scarcity of these resources (Miro et al. 2017).

DSR can be classified as wet DSR, dry DSR, or water DSR (Mahender et al. 2015). Compared with wet and dry DSR, water DSR (whereby seeds are broadcast into standing water) is more advantageous because it is less labour and time intensive and restrains weed growth (Yamauchi et al. 1993). However, rice is extremely sensitive to anoxia during germination and early seedling growth (Yamauchi et al. 1993; Ismail et al. 2009; Yang et al. 2019b). In the case of heavy rainfall during the germination of direct-seeded rice, standing water on the soil surface will significantly affect the seedling rate and thus affect yield (Magneschi et al. 2009). The rice coleoptile is one of the few plant tissues that can grow under hypoxic conditions (Pearce and Jackson 1991). Anoxia promotes coleoptile elongation and inhibits the growth of young stems and roots (Fred et al. 1981). Under anaerobic conditions, the coleoptile elongates rapidly, breaks through the water surface and transports oxygen to the underwater tissues (Alpi and Beevers 1983). The coleoptile elongates rapidly to reach the aerobic environment, providing nutrients for the primary leaves and roots and then promoting seedling morphogenesis. Therefore, it is of great significance to explore the mechanism of rice anaerobic germination (AG) tolerance and the morphological and physiological responses of the coleoptile in anaerobic environments to promote the development of rice direct seeding technology.

When rice is in an anaerobic environment, its coleoptile elongation varies widely among different varieties as a typical quantitative characteristic (Perata and Alpi 1993; Magneschi et al. 2009; Magneschi and Perata 2009). To determine the genetic basis of this phenomenon, in recent years, quantitative trait loci (QTLs) have been used to identify different populations, and some QTLs related to coleoptile elongation have been found. Angaji (2008) reported four putative QTLs on chromosomes 1, 2, 11, and 12 in a BC_2_F_2_ population derived from the cross of KHAIYAN and IR64. These QTLs explained 51.4% of the phenotypic variance. Similarly, Khao Hlan On, a anoxia-tolerant accession, was crossed with IR64 to construct BC_2_F_2_ lines, and five QTLs were found on chromosomes 1, 3, 7, and 9 (Angaji et al. 2009). Among them, *TPP7* (Kretzschmar et al. 2015) was found in *qAG 9–2* (Angaji et al. 2010) and shown to participate in trehalose-6-phosphate metabolism, enhance starch mobilization, and improve AG tolerance. Offspring with the tolerance allele showed a high germination rate, as their seedlings could reach the water surface (Angaji et al. 2010). Septiningsih et al. (2013) used 118 simple sequence repeat (SSR) molecular markers and an F_2:3_ population constructed from IR42 and Ma-Zhan Red to detect 6 QTLs related to seed germination and flooding tolerance under 10 cm flooding stress, and the BC_2_F_3_ generation was used to verify the major effect locus *qAG7.1* on chromosome 7 and estimate its contribution rate at 31.7%. Lee et al. (2017) used backcross inbred lines (BILs) of Kasalath and Nipponbare to evaluate deep sowing (5 cm sowing depth) performance. With coleoptile length (CL) as an indicator, a QTL location assay identified sites that affected coleoptile elongation on chromosomes 3 and 5, and these sites explained phenotypic variations of 11.8% and 12.0%, respectively (Lee et al. 2017). Yang et al. (2019a, 2019b) used linkage analysis of 192 recombinant inbred lines (RILs) produced from YZX and 02428 to detect 13 stable QTLs on chromosomes 1, 2, 3, 4, 6, 7, 9, 10 and 12, taking the CL, coleoptile diameter (CD), coleoptile surface area (CSA) and coleoptile volume (CV) at 6 days after submergence and the germination of the seeds of the two generations as indicators.

Most of the existing reports are based on the genetic site mining of populations derived from different parents (such as RILs; NILs; and BILs), but these studies are limited by the genetic information possessed by the parents and by molecular marker density, so their identification of flooding-resistant germination loci related to coleoptile elongation is limited.

Genome-wide association studies (GWAS) based on SSR (Li et al. 2012) or single nucleotide polymorphism (SNP) markers (Huang et al. 2010; Zhao et al. 2011; Huang et al. 2012) have been widely used in model plants, including rice. By identifying high-density SNPs in different germplasm accessions based on second-generation genome sequencing or SNP array approaches, we can obtain a very high resolution (Chen et al. 2014; Yu et al. 2014; Wu et al. 2015), which provides a strong advantage for the mining of complex QTLs. Hsu and Tung (2015) analysed the association of 153 indica, japonica and Australian rice populations by using genome-wide association technology and found that 88 significant SNPs were correlated with the anaerobic response index based on differences in CL between control and submerged rice plants. Among them, only one unique QTL on the long arm of chromosome 1 showed a strong signal in a RIL population from Nipponbare/IR64 hybridization, and this QTL explained approximately 27% of the phenotypic variation (Hsu and Tung 2015). The study revealed that the *HXK6* gene was located in this genomic region. HXK6 is a double-target mitochondrial and nuclear protein (Cho et al. 2009; Huang et al. 2009). As a glucose sensor (Cho et al. 2009;Granot et al. 2013; Granot et al. 2014), it may be involved in hexose phosphorylation to regulate the expression of important hypoxia-inducible genes, such as *CIPK15* and alcohol dehydrogenase (Yim et al. 2012; Lim et al. 2013; Shingaki-Wells et al. 2014), and therefore improve the survival rate of rice seedlings during submerged germination.

Zhang et al. (2017) carried out GWAS analysis using a mixed linear model with 432 *indica* rice varieties in normal and flooded environments and detected 11 and 9 significant SNPs for flooded coleoptile length (FCL) and flood tolerance index (FTI). Haplotype analysis found that LOC_Os06g03520 contained a protein DUF domain similar to that in genes involved in the energy metabolism pathway (*OsTPP7* and *OsCIPK15*) and was a highly anoxia-induced candidate gene. Nghi et al. (2019) conducted a GWAS on 273 *japonica* rice materials to find new chromosomal regions related to the ability of rice coleoptiles to elongate under flooding conditions. Eleven significant SNPs were found, and 21 candidate genes related to CL were identified.

Coleoptile elongation is the only organic response of rice to anaerobic stress and a reliable characteristic used to study the flooding tolerance of rice genotypes (Setter and Ella 1994; Kato-Noguchi and Morokuma 2007; Zhang et al. 2017). The ongoing improvements of genome sequencing technology will significantly improve the efficiency of GWAS technology in genetic locus identification by allowing researchers to obtain high-density SNPs by sequencing. In this study, phenotypic data (CL and CD) and 2,123,725 SNP markers were used to perform a dynamic GWAS. The purpose of this study was to detect genetic loci significantly associated with AG in rice, to explore favourable SNP alleles that may be used to breed rice suitable for direct seeding and to find reliable candidate genes to lay a foundation for future study of the molecular mechanism of AG in rice.

## Results

### Statistical Analysis of Phenotypic Variation in Anaerobic Germination Tolerance

**Fig. 1:**
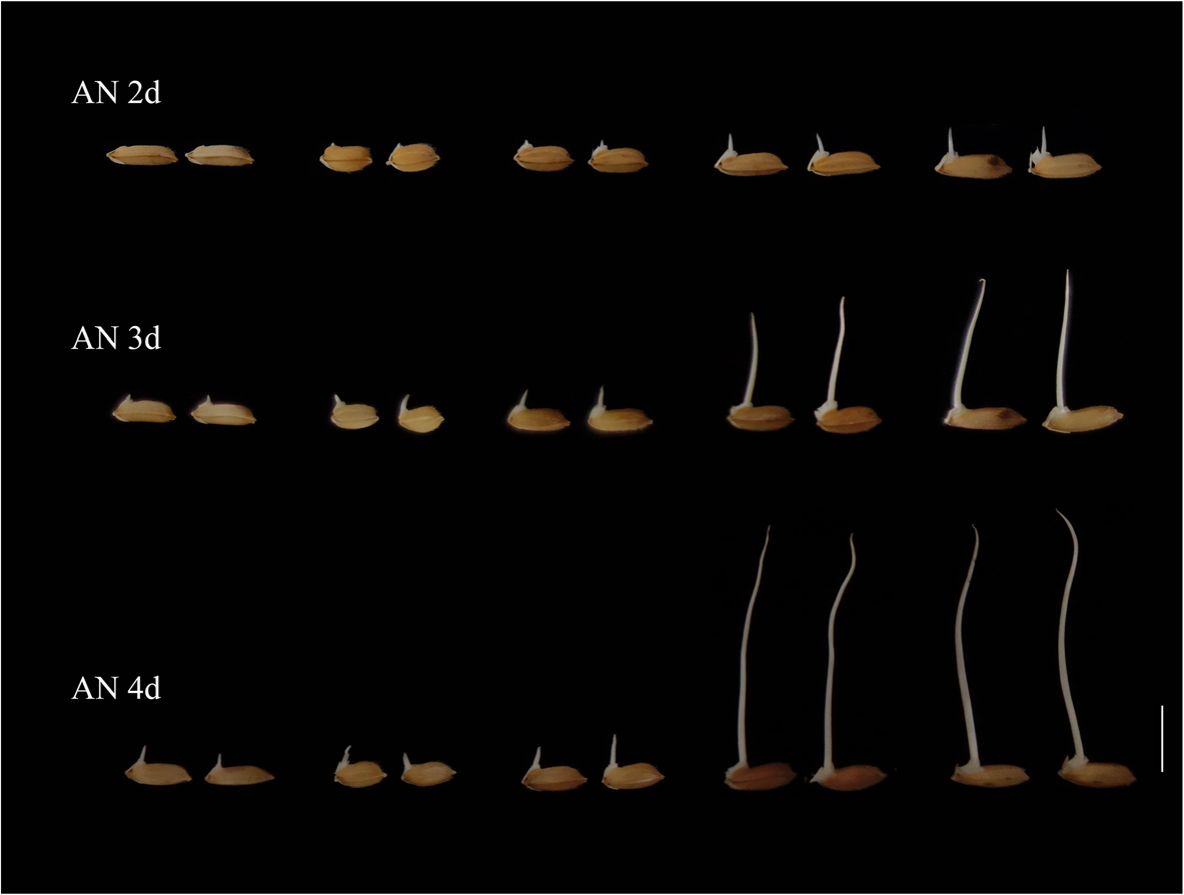
Phenotypic variation of coleoptile in different rice germplasm at different submerging time points. AN is for anaerobic environment. Scale = 1 cm

A total of 209 *indica* and *japonica* rice accessions from all over the world (Table S1) were tested for anaerobic germination. CL, CD, CSA and CV on the second, third and fourth days of germination were used as the indexes of submergence tolerance. Abundant genetic variation was detected in this *indica* and *japonica* rice accession population (Fig. 1, Table S2). Analysis of variance showed that the 209 rice accessions had significant differences in submerged coleoptile phenotype at different time points. At the same time, both genotype and length of submergence time had significant influences on coleoptile phenotype (Table 1). The variation coefficients of CL, CSA and CV were greater than 10% on the 2nd, 3rd and 4th days of submergence, indicating that the CL, CSA and CV of the different varieties were significantly different. The variation coefficients of CL, CD, CSA and CV were the greatest on the second day of submerging. With increasing submerging time, the variation coefficients of each phenotype decreased gradually, indicating that the differences in each phenotype were significant on the second day of submerging, and the coleoptiles grew rapidly to avoid the anaerobic environment.

**Table 1:** Phenotypic variation of rice coleoptile during submergence germination

Phenotype	Environment	Mean ± SE	Range	Coefficient of variation (%)	P × E
CL	AN2d	0.2226 ± 0.0132 cm	0.0062 ~ 0.6463 cm	18.7571	***^a^
	AN3d	0.6629 ± 0.0258 cm	0.0334 ~ 1.7719 cm	13.0543	
	AN4d	1.2068 ± 0.0498 cm	0.1659 ~ 3.8798 cm	12.9767	
CSA	AN2d	0.0266 ± 0.0051 cm^2^	0.0045 ~ 0.6463 cm^2^	56.1438	
	AN3d	0.1104 ± 0.0048 cm^2^	0.0098 ~ 0.3259 cm^2^	14.3195	
	AN4d	0.2030 ± 0.0089 cm^2^	0.0201 ~ 0.7689 cm^2^	13.7986	
CD	AN2d	0.4543 ± 0.0272 mm	0.1092 ~ 0.6597 mm	22.4859	
	AN3d	0.5155 ± 0.0137 mm	0.3908 ~ 0.6837 mm	7.9094	
	AN4d	0.5496 ± 0.0065 mm	0.4452 ~ 1.0680 mm	3.5583	
CV	AN2d	0.0008 ± 0.0001 cm^3^	0.0001 ~ 0.0625 cm^3^	35.6686	
	AN3d	0.0015 ± 0.0001 cm^3^	0.0049 ~ 0.0015 cm^3^	18.3201	
	AN4d	0.0030 ± 0.0010 cm^3^	0.0003 ~ 0.0419 cm^3^	20.8486	

^a^ *** indicates a significant correlation between phenotype and environment treatment.

**Fig. 2:**
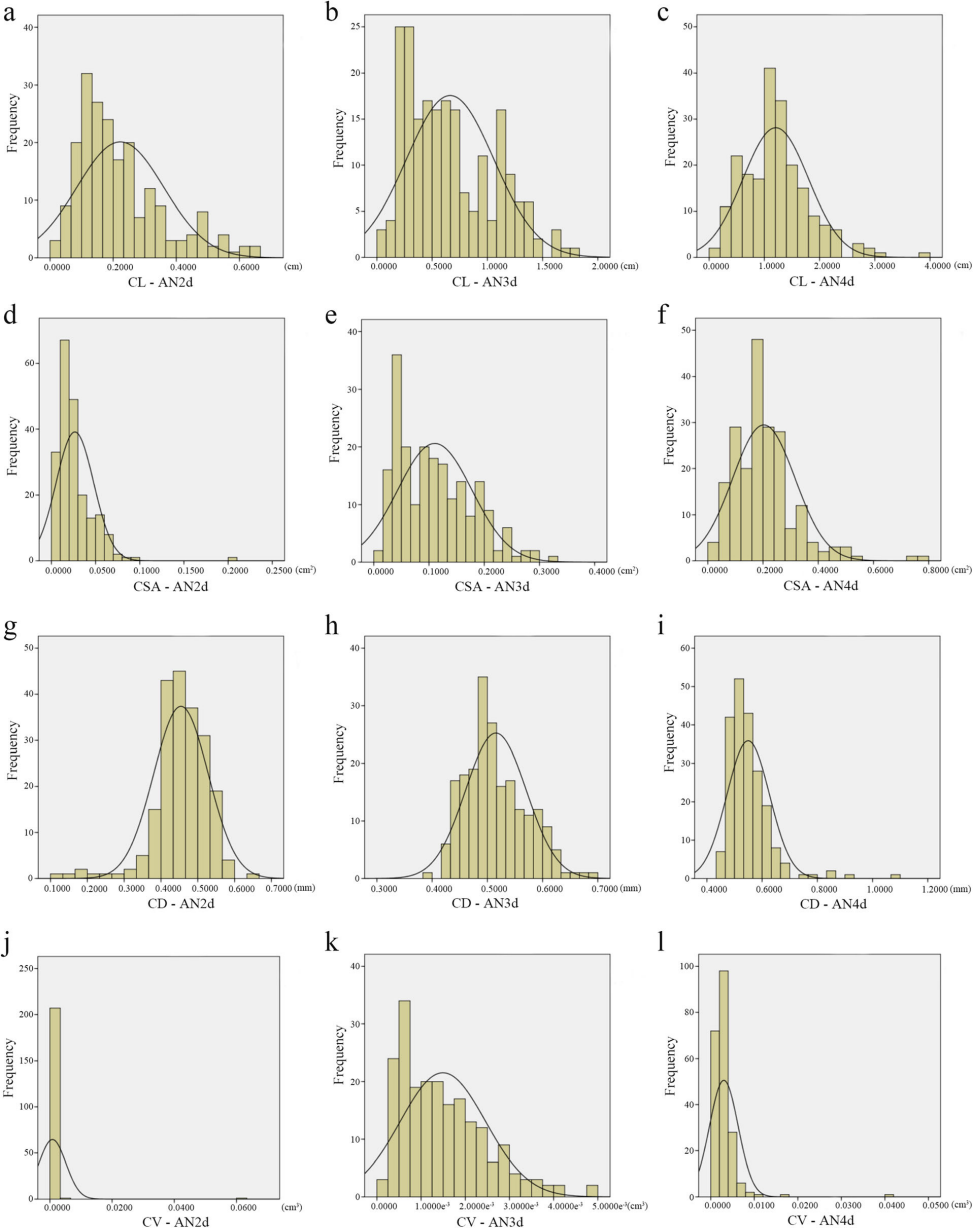
Frequency distribution histogram of each phenotype of coleoptile at different submergence time points. Note: CL, coleoptile length; CSA, coleoptile surface area; CD, coleoptile diameter; CV, coleoptile volume

It can be seen from the histogram of frequency distribution (Fig. 2) that after 4 days of continuous oxygen-deficiency treatment, the AG tolerance indexes were consistent with a normal distribution (except CV at 2 and 4 days of submergence), indicating that these traits are regulated by many genes with small effects.

### Correlation Analysis of Phenotypic Traits

Correlation analysis showed correlations among the coleoptile phenotypes after 2 days, 3 days and 4 days of submergence stress (Table 2). Among them, CV-AN2d/CL-AN2d and CSA-AN2d/CSA-AN3d/CSA-AN4d/CD-AN2d/CD-AN3d were correlated, but there was no correlation between CV-AN4d and CD-AN2d. CV-AN2d was negatively correlated with CL-AN3d, CL-AN4d, CD-AN4d, CV-AN3d and CV-AN4d. CL-AN3d/CSA-AN3d/CV-AN3d, CL-AN4d/CSA-AN4d, and CSA-AN3d/CV-AN3d had very significant positive correlations with a *P*-value> 0.800.

**Table 2:** Correlation analysis of coleoptile phenotpye

Trait	CL - AN2d	CL - AN3d	CL - AN4d	CSA -AN2d	CSA - AN3d	CSA - AN4d	CD - AN2d	CD - AN3d	CD - AN4d	CV - AN2d	CV - AN3d	CV- AN4d
CL - AN2d	1											
CL - AN3d	0.521^a^	1										
CL - AN4d	0.475^a^	0.739^a^	1									
CSA -AN2d	0.767^a^	0.442^a^	0.405^a^	1								
CSA - AN3d	0.567^a^	0.948^a^	0.725^a^	0.385^a^	1							
CSA - AN4d	0.445^a^	0.657^a^	0.912^a^	0.403^a^	0.668^a^	1						
CD - AN2d	0.290^a^	0.268^a^	0.291^a^	0.304^a^	0.353^a^	0.250^a^	1					
CD - AN3d	0.215^a^	0.180^a^	0.189^a^	0.266^a^	0.313^a^	0.293^a^	0.632^a^	1				
CD - AN4d	0.226^a^	0.148^b^	0.181^a^	0.435^a^	0.195^a^	0.272^a^	0.404^a^	.729^a^	1			
CV - AN2d	0.066	−0.034	−0.023	0.128	0.042	0.002	0.054	0.005	−0.01	1		
CV - AN3d	0.549^a^	0.885^a^	0.710^a^	0.463^a^	0.924^a^	0.687^a^	0.394^a^	0.452^a^	0.354^a^	−0.032	1	
CV- AN4d	0.217^a^	0.296^a^	0.436^a^	0.205^a^	0.324^a^	0.730^a^	0.01	0.204^a^	0.175^b^	−0.016	0.409^a^	1

^a^At 0.01 level (2-tailed), the correlation was significant

^b^At 0.05 level (2-tailed), the correlation was significant

The dynamic test results of coleoptile phenotypes showed significant correlations among CL and CD measurements at various time points, and these traits play key roles in seed tolerance of anaerobic conditions. This conclusion was also supported by previous work. Therefore, GWAS analysis was performed with CL and CD as phenotypes.

### Whole-Genome Resequencing and Polymorphism Identification

In this study, the genomes of 209 rice germplasm accessions were resequenced. Burrows-Wheeler Aligner (BWA) software was used to compare the sequencing data with the reference genome IRGSP-1.0 (Kawahara et al. 2013). Finally, 1243.37 Gbp of clean data was obtained, and the Q30 reached 93.15%. The average contrast ratio between each sample and the reference genome was 93.06%, the average sequencing depth was 12 ×, and the average coverage of the reference genome was 90.90% (at least one base coverage).

A total of 2,123,725 SNP sites were detected in this dataset. Among them, the SNP types were mainly C:G > T:A and T:A > C:G (Fig. S1a). Using SnpEff software to annotate the detected SNPs, we found 663,818 SNPs with synonymous mutations and 712,374 with nonsynonymous mutations (Fig. S1b).

### Population Genetics and Evolutionary Analysis

#### Phylogenetic and Population Structure Analysis

**Fig. 3:**
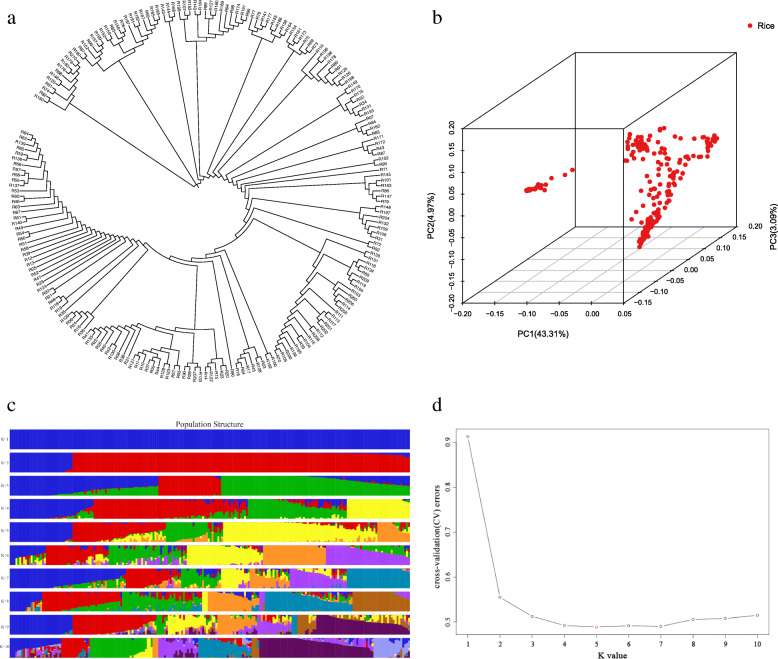
Genetic evolution of natural populations of *indica* and *japonica* rice. **a** Phylogenetic tree, each branch is a rice germplasm. **b** Principal component analysis on 2.12 million SNPs of 209 rice accession. PC 1 and PC 2 refer to the first and second principal components, respectively. The numbers in parentheses refer to the proportion of variance explained by the corresponding axes. Red points represent each variety in 209 rice accession. Each point represented a rice germplasm accession. The closer the distance between the points, the closer the relationship was. **c** Cluster analysis results of population genotypes, in which each color represents a group and each row represents a group value. **d** Cross validation error rate for each k value. Among them, K is the smallest when k is 5

From the phylogenetic tree (Fig. 3a), it can be seen that the population structure used in this experiment is uniform, without strong population stratification. Based on the SNPs, Admixture (Alexander et al. 2009) software was used to analyse the group structure of the research materials. Cross-validation error analysis showed that the error peak was lowest at K = 5, indicating the optimal grouping (Fig. 3c). The population structure analysis showed no obvious pedigree differentiation in the selected plant materials, confirming that they were suitable for subsequent GWAS analysis. The phylogenetic tree results showed that the selected population could be divided into 5 subgroups, which verified the conclusion that K = 5 was the optimal result in the population structure analysis. The Q matrix with K = 5 was selected for the subsequent association analysis (Fig. 3d).

Based on the SNP data, EIGENSOFT software was used to perform principal component analysis (PCA) (Price et al. 2006) to cluster the samples (Fig. 3b). The results of PCA supported the evolutionary analysis, further confirming that the degree of discreteness of individual kinship in the population was small.

### Dynamic Genome-Wide Association Analysis of Coleoptile Phenotypic Traits under Submergence

**Fig. 4:**
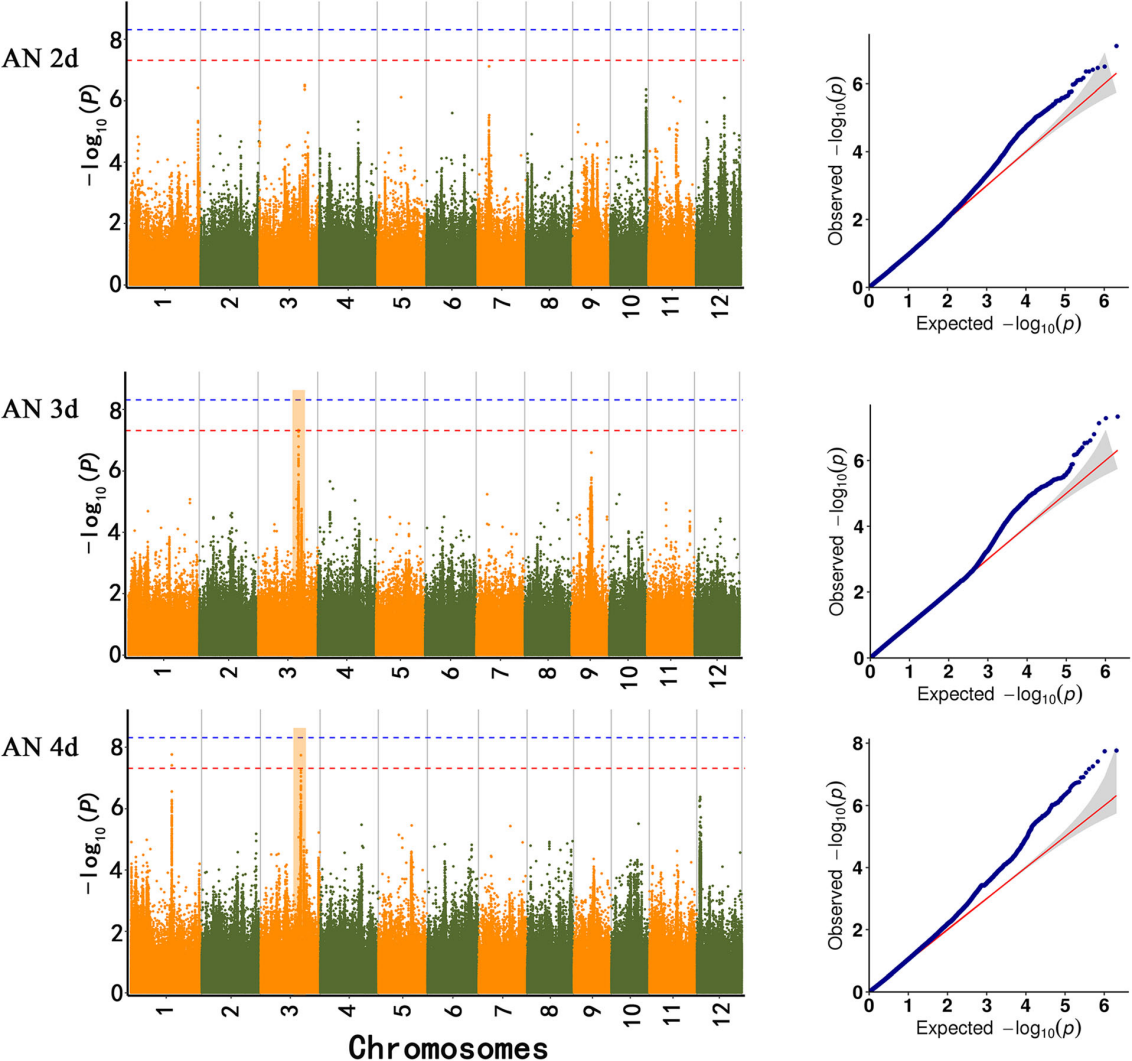
Manhattan map of SNPs associated with coleoptile length at different time points under anaerobic germination. The red horizontal line indicates the significance threshold of multiple comparisons (*P* < 0.1). The genomic regions detected in our GWAS and previous parental mapping studies were labeled as published QTLs with blue text. Orange blocks represented two co-localized genomic regions under anoxic pressure for different lengths of time in our GWAS study

**Fig. 5:**
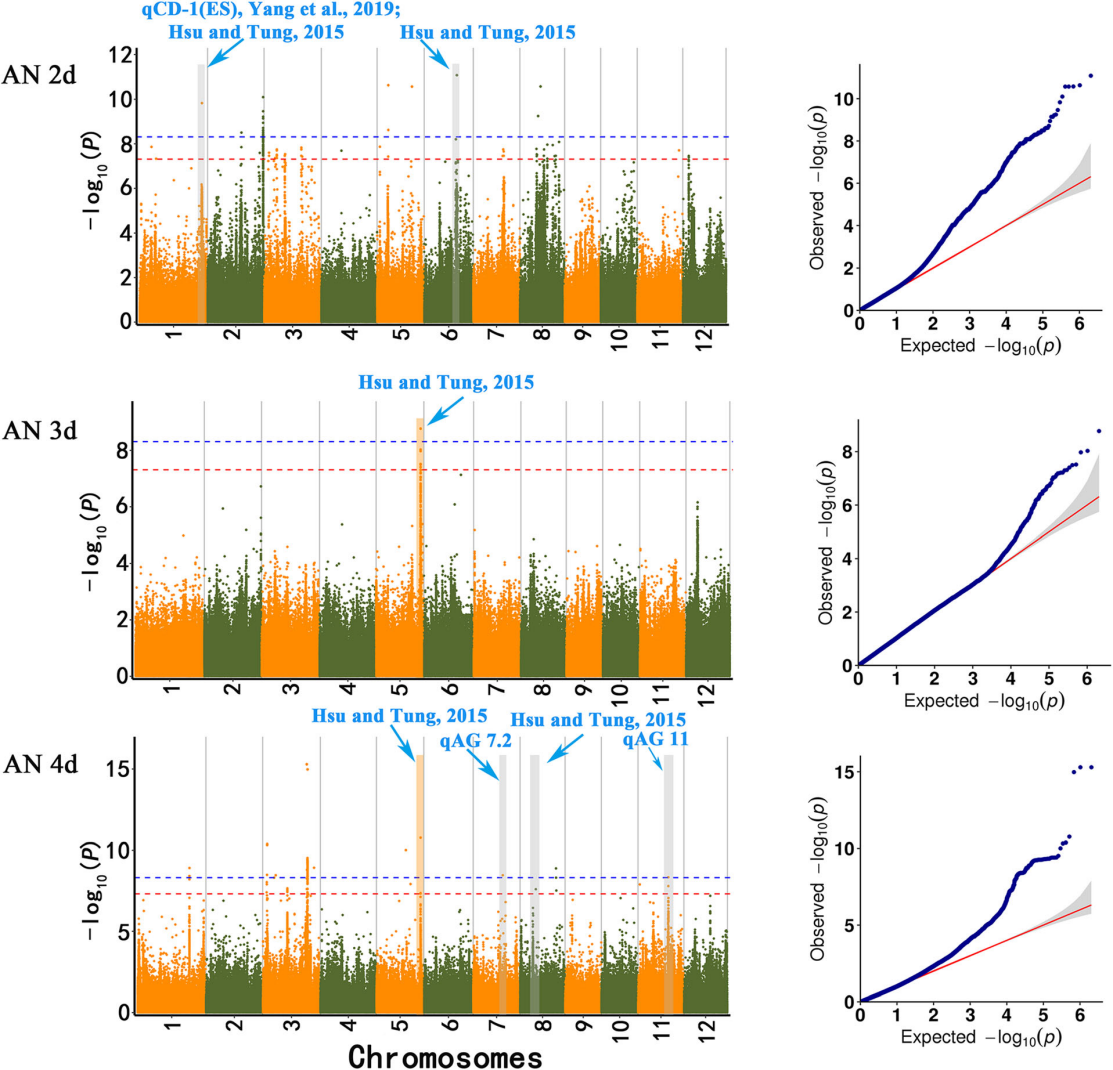
Manhattan map of SNPs associated with coleoptile diameter at different time points under anaerobic germination. The red horizontal line indicates the significance threshold of multiple comparisons (*P* < 0.1). The genomic regions detected in our GWAS and previous parental mapping studies were labeled as published QTLs with blue text. Orange blocks represented two co-localized genomic regions under anoxic pressure for different lengths of time in our GWAS study

Based on the developed high-density SNP marker data, we performed a GWAS on the dynamic changes in two phenotypic traits, CL and CD, to mine new genetic loci associated with AG. The Manhattan plots and quantile-quantile (QQ) plots are shown in Figs. 4 and 5. Taking −log_10_ (*P*) value > 7.3271 as the threshold, CL was not associated with GWAS sites under AN2d condition; CD was associated with 23 GWAS sites, including 29 SNPs with extremely significant correlation. Under AN3d condition, CL is associated with one GWAS site, which contains one extremely significant SNP, namely S3_ 24689629. CD is associated with one GWAS site and contains 7 SNPs that are significantly related. Under AN4d condition, CL was associated with 2 GWAS site, including 3 SNPs with extremely significant correlation. CD is associated with 16 GWAS site, including 146 SNPs that are significantly correlated (Table S9). According to the values of LD, carefully consider the SNPs in the same area of LD detected after we finalized the 22 significant SNPs associated with CD (8 at AN2d, 1 at AN3d, and 14 at AN4d) and were distributed on chromosomes 1, 2, 3, 5, 6, 7, 8, and 11. Two SNPs were significantly correlated with CL (0 at AN2d, 1 at AN3d, and 2 at AN4d), and they were distributed on chromosomes 1 and 3 (Table 3).

**Table 3:** Genome-wide significant associations for coleoptiles phenotype under anaerobic germination (AG) using MLM

Traits	SNP^a^	Chr.	Peak position^b^	*P* value	—log10 (*P*)	Allleles	MAF	Known loci
CD-AN2d	S1_ 39674301	1	39674301	1.48E-10	9.83	A/C	0.2	Hsu and Tung 2015; *qCD-1*(ES) (Yang et al. 2019a, 2019b)
	S2_ 21568963	2	21568963	3.14E-09	8.5	C/T	0.2	
	S2_ 35360509	2	35376690	7.98E-11	10.1	A/G	0.2	
	S5_ 7135836	5	7135836	2.32E-11	10.63	C/T	0.1	
	S5_ 22190175	5	22190175	2.69E-11	10.57	G/A	0.2	
	S6_ 20797781	6	20797781	8.49E-12	11.07	G/A	0.1	Hsu and Tung 2015
	S8_ 13123867	8	13123867	2.65E-11	10.58	G/T	0.1	
	S8_ 11595732	8	11595732	5.64E-10	9.25	A/T	0.1	
CD-AN3d	S5_ 27918754	5	27918754	1.69E-09	8.77	A/G	0.4	Hsu and Tung 2015
CD-AN4d	S1_ 32866906	1	32866906	1.24E-09	8.91	G/A	0.2	
	S3_ 27854371	3	27854371	5.11E-16	15.29	A/G	0.3	
	S3_ 2828271	3	2828271	4.16E-11	10.38	A/G	0.2	
	S3_ 28290634	3	28290634	3.02E-10	9.52	G/A	0.1	
	S3_ 32459722	3	32459722	1.20E-09	8.92	C/T	0.5	
	S3_ 7243651	3	7243651	4.89E-09	8.31	C/A	0.2	
	S3_ 15571447	3	15571447	2.23E-08	7.65	G/T	0.3	
	S5_ 27918754	5	27918754	1.72E-11	10.76	A/G	0.4	Hsu and Tung 2015
	S5_ 18621890	5	18621890	9.76E-11	10.01	A/C	0.1	
	S5_ 21585755	5	21585755	1.21E-08	7.92	T/A	0.2	
	S7_ 18722403	7	18722403	3.45E-09	8.46	T/C	0.2	*qAG 7.2* (Septiningsih et al. 2013; Hsu and Tung 2015)
	S8_ 22721012	8	22721012	1.31E-09	8.88	A/G	0.2	
	S8_ 9946213	8	9946213	2.51E-08	7.6	T/A	0.2	Hsu and Tung 2015
	S11_ 19165397	11	19165397	4.62E-09	8.34	C/A	0.3	*qAG 11*(Jiang et al. 2006; Angaji et al. 2009; Zhang et al. 2017)
CL-AN3d	S3_ 24689629	3	24689629	4.71E-08	7.33	T/C	0.4	
CL-AN4d	S1_ 25099585	1	25099585	1.73E-08	7.76	G/A	0.2	
	S3_ 24689629	3	24689629	1.83E-08	7.74	T/C	0.4	

Note: MAF minor allele frequency

^a^The SNP positions were based on the annotation data on Reference genome IRGSP-1.0 (RAP-DB, http://rapdb.dna.affrc.go.jp/)

^b^Position of the SNP showing the most significant association for AG

**Fig. 6:**
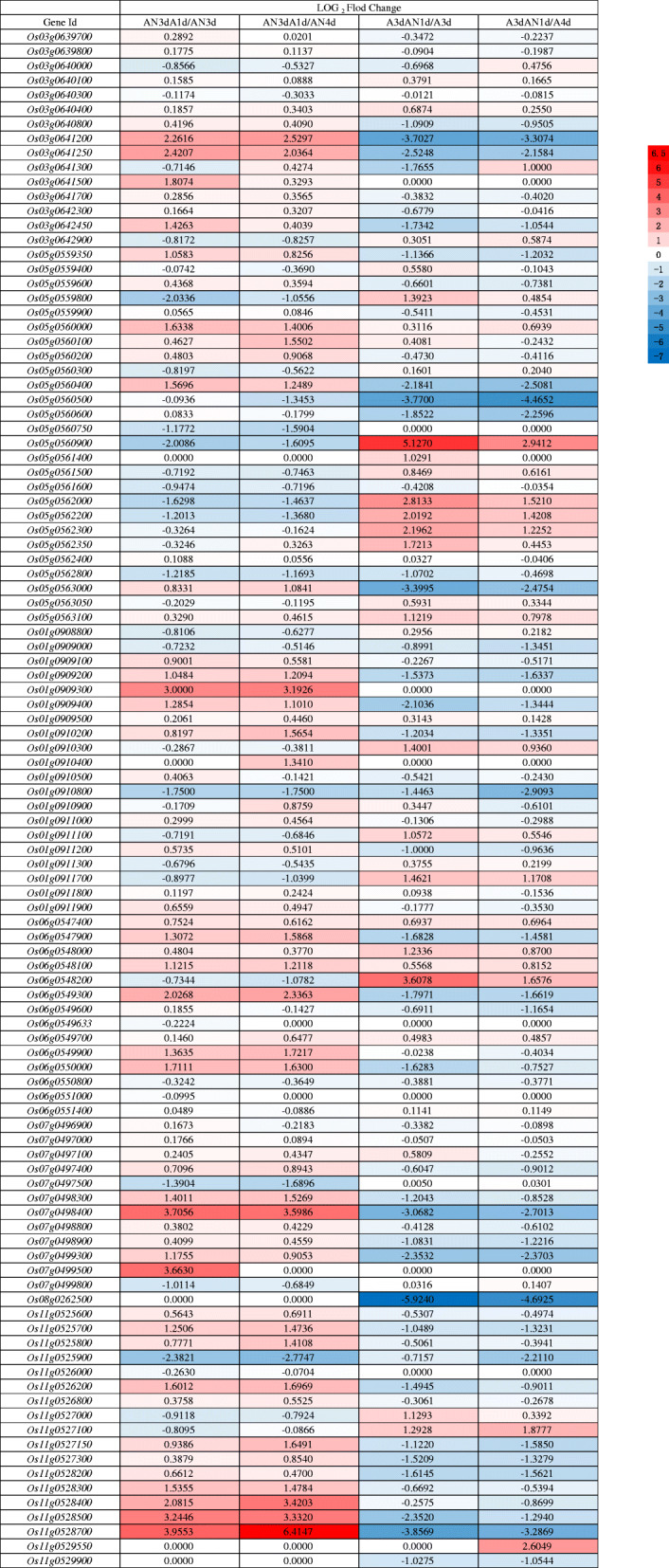
Heat map of the fold changes of the 106 candidate differentially expressed genes (DEGs)

To verify the accuracy of our results, we compared them with previously reported QTLs (Jiang et al. 2006; Yang et al. 2019a, 2019b) related to the control of coleoptile elongation and the survival rate of plants under flooding conditions (Angaji et al. 2010; Baltazar et al. 2014; Septiningsih et al. 2013). We found that some of our QTLs has been identified before in rice submerged germination tolerance. We found that 6 genomic regions were colocalized (grey and orange blocks in Fig. 6). A SNP on chromosome 1 (S1_ 39674301), which was significantly associated with CD in this study, was located in the *qCD-1*(ES) gene interval reported by Yang et al. (2019a, 2019b) as closely related to CD during the early cropping season. One SNP (S6_20797781) located on chromosome 6 was very close to a gene locus detected in another association study (Hsu and Tung 2015). The physical distance between these two SNPs was only approximately 4 kb. Another SNP (S7_18722403) significantly correlated with CD at AN4d was found inside the genomic interval of *qAG7.2* on chromosome 7; this locus was previously detected using linkage mapping and association mapping (Septiningsih et al. 2013; Hsu and Tung 2015), validating its genetic effect. On chromosome 8, a SNP (S8_ 9946213) significantly associated with CD was very close to a site detected in another related study as closely related to the anaerobic response index (treated CL vs. control CL; Hsu and Tung 2015), and the physical distance between them was approximately 95 kb. On chromosome 11, another SNP (S11_ 19165397) significantly associated with CD was located in the genomic region of *qAG 11*, which was closely related to the submerged CL (Hsu and Tung 2015) and the anaerobic response index (*qAG 11*, Jiang et al. 2006; Angaji et al. 2009).

In addition, some loci were identified at multiple anoxia time points. CD was correlated with SNP S5_ 27918754 at AN3d and AN4d (Fig. 5), as reported in a previous study (Hsu and Tung 2015). In addition, CL was corelated with SNP S3_24689629 (Fig. 4) at AN3d and AN4d, and this result had not been reported previously.

### Identification of Candidate Genes

We selected 7 loci from the GWAS results, including S3_ 24689629 and S5_ 27918754, which were repeatedly detected for CD and CL, respectively, at both AN3d and AN4d, indicating that these two loci were highly correlated with coleoptile elongation and thickening under hypoxic conditions. In addition, S1_39674301, S6_20797781, S7_18722403, S8_9946213, and S11_19165397 (Hsu and Tung 2015; Yang et al. 2019a, 2019b; Septiningsih et al. 2013; Zhang et al. 2017; Jiang et al. 2006; Angaji et al. 2009) were consistent with previous research results and were included. Based on the linkage disequilibrium (LD) decay distance (100–350 kb) (Fig. S2), we detected a total of 106 differentially expressed genes (DEGs) in these seven trait-associated SNP sites. To more effectively identify candidate genes related to AG in rice seeds, RNA-seq analysis was carried out on RNA extracted from germinated seeds of the flooding-tolerant variety YZX at AN0d, AN2d, AN3d, AN4d, AN3dA1d, and A3dAN1d. We normalized the FPKM values of these DEGs as follows (Fig. 6): log_2_ FC (AN3dA1d/AN3d), log_2_ FC (AN3dA1d/AN4d), log_2_ FC (A3dAN1d/A3d), and log_2_ FC (A3dAN1d/A4d). Based on the candidate gene selection criteria | log_2_ FC | ≥ 1, FDR ≤ 0.05 and FPKM ≥1 (special: log2 FC (AN3dA1d/AN3d), log_2_ FC (AN3dA1d/AN4d) ≤ 1; log_2_ FC (A3dAN1d/A3d), and log_2_ FC (A3dAN1d/A4d) ≥ 1), a total of 4 DEGs were obtained.

**Fig. 7:**
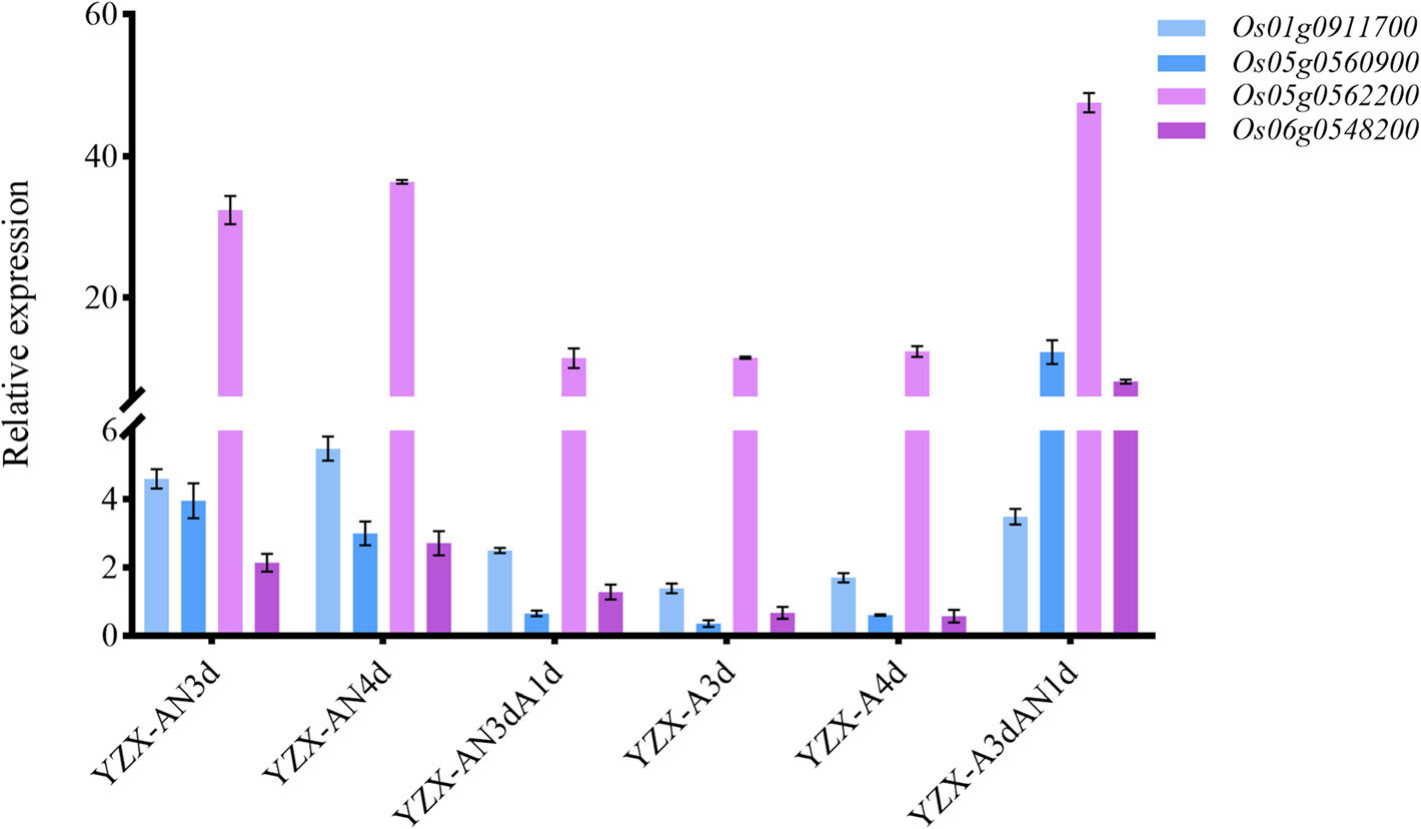
qRT-PCR expression profile of four most promising candidate genes in different oxygen environments. Values are the means ± standard errors (*n* = 3). A is for aerobic environment, AN is for anaerobic environment, AN3dA1d is for anaerobic environment and A3dAN1d is for aerobic environment and anaerobic environment

According to the gene annotation information of the rice reference genome, we concluded that these four DEGs, gibberellin 2-beta-dioxygenase (*OsGA2ox8*, *Os05g0560900*, S5_ 27918754), berberine bridge enzyme-like 13 (*Os06g0548200*, S6_20797781), protein dehydration-induced 19 (*OsDi19–1*, *Os05g0562200*, S5_ 27918754), and B3 domain-containing protein VP1 (*OSVP1*, *Os01g0911700*, S1_39674301), had major impacts on AG in rice seeds. The results of qRT-PCR (Fig. 7, Table S7) were similar to the results of RNA-seq. These four genes were specifically expressed under hypoxic conditions, their expression levels increased with increasing hypoxia time, and their expression levels decreased after transfer to aerobic conditions. In addition, we also referred to the transcriptome data of Lasanthi-Kudahettige et al. (2007) (Table S8), we found that the expression levels of these four genes in coleoptile of anoxia for 4 days were higher than that under aerobic conditions, which indicated that these four genes were induced by anoxia and participated in seed germination. These results indicated that these four DEGs had strong correlations with submerged germination in rice.

### Four New Genes Related to the Anaerobic Germination Phenotype

**Fig. 8:**
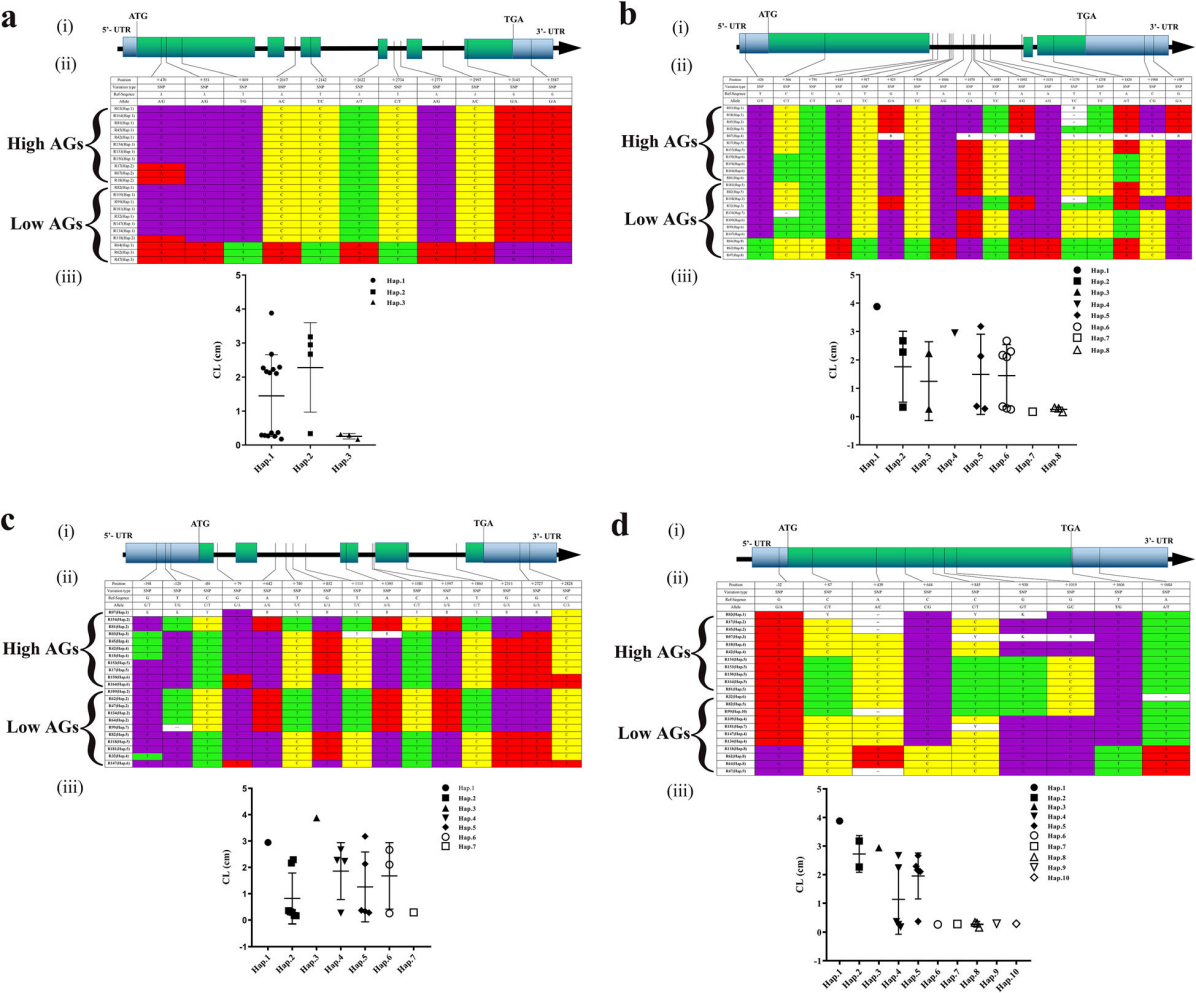
Sequence analysis of four candidate genes in rice with high and low AGs. **a** The positions of four candidate genes *Os01g0911700*, *Os05g0560900*, *Os05g0562200* and *Os06g0548200* on chromosomes. **b** Sequence analysis of candidate gene *Os01g0911700*. **c** Sequence analysis of candidate gene *Os05g0560900.* **d** Sequence analysis of candidate gene *Os05g0562200*. **e** Sequence analysis of candidate gene *Os06g0548200*. Notes: (i) the gene structure of four candidate genes is shown respectively, the blue region represents 5′ or 3′ UTR, the green region represents exon, the straight line represents intron, and the arrow shows gene direction. (ii) the detailed sequence changes of four candidate genes in rice materials with very high and very low AGs are shown, where the position number represents the position related to the “ATG” initiation codon; “-” represents the upstream of “ATG”; and “+” represents the downstream of “ATG”. “--” means that the gene is missing 1 bp. (iii) The phenotypic values of each haplotype

To further verify the associations between the candidate genes and the germinating coleoptile phenotype under AG conditions, we performed a full-length sequencing analysis of the four candidate genes in materials with extreme phenotypes (11 high-AG materials and 11 low-AG materials) (Fig. 8).

*Os01g0911700* (*OsVP1*) encodes the VP1 protein, which contains the B3 domain and is a key transcription factor in the abscisic acid (ABA) signal transduction pathway. Among the 11 high-AG materials and 11 low-AG materials, we identified 11 allelic variants and 3 haplotypes of this gene (Fig. 8a). Of the 11 allelic variations, 10 included mutation sites within the gene, and these sites were mainly distributed in the first, third and sixth exon regions. The 11 high-AG varieties were different from the low-AG varieties at these 10 loci. The haplotype analysis of the extreme materials showed that Hap.2 (compared to Hap.3, all the variable sites except + 470, downstream of the start codon, were different) was associated with the high-AG phenotype based on the traits of the coleoptile at AN4d. The average CL value was 2.932 cm. Plants carrying Hap.3 (in which none of the 11 variable sites were mutated) all had the low-AG phenotype, and the average CL value at AN4d was 0.257 cm (Table S5). we conjecture that the mutations at the remaining 10 sites have a greater impact on the AG phenotype than the mutation at site + 470 (downstream of the start codon). Among them, + 3143 is located in the highly conserved C-terminal B3 domain, coding for aa (D); + 470, + 551, + 809, + 2142 are synonymous mutations, encoding aa (D), aa (D), aa (A), aa (E); + 2017, + 2622, + 2734, + 2771, + 2997 are non coding region variants. + 3587 is located in the 3’UTR region. It is A (reference sequence) in high-AG haplotype and G (mutation type) in low-AG haplotype.

The *Os05g0560900* (*OsGA2ox8*) gene encodes a gibberellin 2-beta-dioxygenase, which is involved in GA catabolism. From the gene sequences of the 11 high-AG materials and 11 low-AG materials, we identified 17 allelic variations (Fig. 8b), which were mainly located in the first intron region. Two were located in the first and third exon regions, and the first was located at position 366 (C → T) after the start codon, causing a missense mutation of glycine (G) to serine(S). The second was located at position 1420 (A → T) after the start codon, causing a missense mutation from glutamine (Q) to leucine (L). They are both located in low complexity domains, not important functional regions of proteins encoded by OsGA2ox8, and may have less impact on the function of the proteins they encode. Haplotype analysis showed 8 haplotypes in these 22 extreme materials (Table S5). Among them, Hap.2 was associated with the high-AG phenotype. From the perspective of gene sequence, the high-AG phenotype appeared to be caused by a heterozygous mutation or deletion at + 1170 (downstream of the start codon) and was apparent in seedlings at AN4d, when the average CL was 2.931 cm. Hap.5 and Hap.6 had missense mutations in the first exon (+ 366) and the third exon (+ 1420). Because the mutations at these two sites do not fall in important functional regions of the proteins that code for OsGA2ox8, plants with these haplotypes showed both high-AG and low-AG phenotypes, and their average CL values at AN4d were 2.412/2.274 and 0.296/0.284 cm, respectively. In contrast, Hap.8 was associated with the low-AG phenotype, and the average CL value of plants with this haplotype at AN4d was 0.257 cm (Table S5). In addition, 12 of the 17 SNPs were concentrated in the first intron region. Noncoding RNA regions can participate in gene expression regulation in various forms, including tRNA, rRNA, small nuclear RNAs (snoRNA), small nuclear RNA (snRNA), microRNA (miRNA), long noncoding RNA (lncrna), and pseudogene (Mayr 2015). Hap. 2 and Hap. 8 with extreme phenotype showed different genotypes at these 12 loci, which may imply that the mutations in these non coding regions also have potential effects on seed germination under submerging stress.

*Os05g0562200* (*OsDi19–1*) encodes the dehydration-induced Dil9 protein, which contains two zf-Di19 and one Di19_C domains. From the gene sequences of 22 (11 high-AG and 11 low-AG) materials, we identified 15 allelic variants, all of which were located within the gene (Fig. 8c). The gene sequences of the low-AG material were mainly similar to the sequence of the reference genome, and the gene sequences of the high-AG material harboured different mutations at these 15 loci. We speculated that these 15 allelic variations were closely related to the high-AG phenotype. In particular, T → C at the position of + 1860 caused a missense mutation, which was located in the C-terminal domain of Di19. Its SIFT score is 0.2 (< 0.5). SIFT Score showed that this SNP had a great impact on the function of proteins. Haplotype analysis showed 7 haplotypes among these 22 extreme materials (Fig. 8c). Hap.2 was the reference genome sequence, and Hap.2 plants mainly showed the low-AG phenotype, with an average CL value at AN4d of 0.288 cm. Hap.4 plants mainly showed the high-AG phenotype, the average CL values were 2.391 cm (Table S5). C-terminal domain of Di19, a protein that increases the sensitivity of plants to environmental stress, such as salinity, drought, osmotic stress and cold. The protein is also induced by an increased supply of stress-related hormones such as abscisic acid ABA and ethylene (Li et al., 2010). High-AG material + 1860 position is mutant base C, while low-AG material is T. We hypothesized that the mutation at this site might have an effect on the germination tolerance of different rice germplasm to anaerobic germination.

*Os06g0548200* encodes berberine bridge enzyme (BBE). We selected 11 high-AG and 11 low-AG materials and identified 9 allelic variations in their gene sequences (Fig. 8d) that were potentially related to the AG phenotype. One missense mutation was located 87 bases downstream of the start codon (C → T, alanine (A) to threonine (T)), which was located in FAD-binding domain. And its SIFT (Sorting Intolerant From Tolerant) score is 0.6 (> 0.5). A missense mutation was located 439 bases downstream of the start codon (C → A, leucine (L) to arginine (R)). It was located in N-terminal of FAD linked oxidase, its SIFT score is 0.51(> 0.5). A missense mutation was located 644 bases downstream of the start codon (C → G, glutamic (E) to aspartic (D)), it was also located in N-terminal of FAD linked oxidase. But its SIFT score is 1 (> 0.5), it shows that it has little effect on the function of coding protein. A missense mutation (T → G) located 930 bases downstream of the start codon resulted in an aa substitution from arginine (R) to serine (S). It is not in the important functional region of the protein encoded by *Os06g0548200*. However, its SIFT score is 0.08 (< 0.5). SIFT Score showed that this SNP was harmful and had a great impact on the function of proteins. In particular, − 32, + 644, + 1607 and + 1685 had only one genotype (mutant type) in the high-AG materials, while there were two different genotypes (mutant and reference genotypes) in the low-AG materials, and these had polymorphisms located in the UTR and outside the exon regions. Haplotype analysis showed 10 haplotypes in these 22 extreme materials (Table S5), of which Hap.5 showed a high-AG phenotype, and its average CL at AN4d was 2.274 cm. Hap.8 showed a low-AG phenotype, and its average CL at AN4d was 0.273 cm (Table S5). Hap.5 had mutations at − 31, + 645, + 1607, and + 1685, and Hap. 8 had the opposite pattern, so we believe that these four sites may be closely related to the high-AG phenotype.

### Metabolic Spectrum Detection of Rice Seeds under Anaerobic Germination

LC-MS/MS was used to obtain the mass spectrum data of samples, and qualitative and quantitative analysis was carried out on the basis of a self-established database (MWDB) by Metware Company and public metabolite information database. In total, 730 metabolites were identified, including 32 substances and their derivatives (Table S3).

**Fig. 9:**
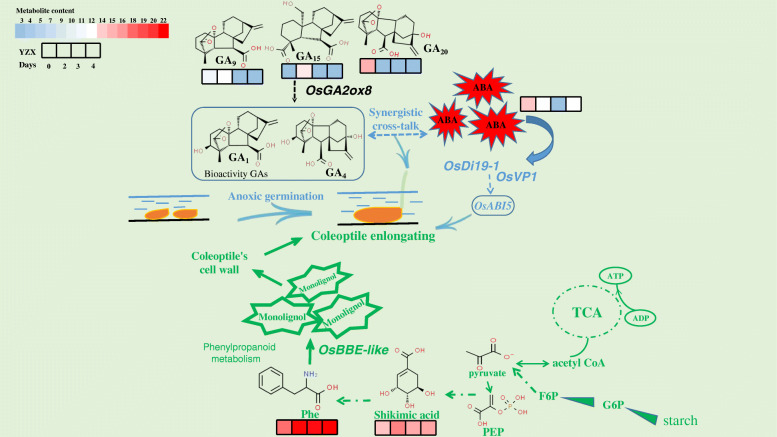
Proposed mechanism of four candidate genes function in enhancing anaerobic germination. Abbreviations are as follows: G6P, Glucose-6-phosphate; F6P, Fructose-6-phosphate; PEP, phosphoenolpyruvic acid; Phe, phenylalanine; TCA, tricarboxylic acid cycle

**Fig. 10:**
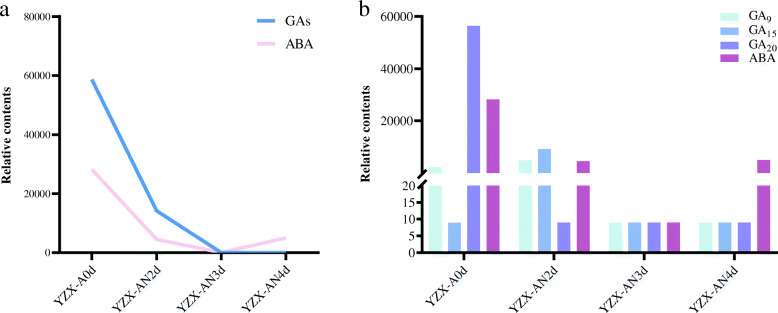
**a** The relative content of gas and ABA in YZX seeds at different submerging time points. **b** The relative contents of three kinds of GA: GA_9_/GA_15_/GA_20_ and ABA under the condition of anaerobic germination. AN is for anaerobic environment

Through the detection of metabolites in the seeds of the YZX rice variety during AG, we found that the contents of metabolites regulated by the four genes identified above were specifically correlated with hypoxic stress. Among them, we detected three biologically active GAs, GA_9_, GA_15_, and GA_20_, which are related to the regulation of GA catabolism by the *OsGA2ox8* (*Os05g0560900*) gene. The contents of GA_9_ and GA_15_ increased within 2 days after submergence, and the content at AN4d decreased compared to that at AN3d; the content of GA_20_ decreased to its lowest value within 2 days after submerging and then did not change (Table S3, Fig. 10). *OsVP1* (*Os01g0911700*) and *OsDi19–1* (*Os05g0562200*) participated in the regulation of ABA-related phenotypes. Quantitative results showed that ABA content decreased within 3 days after submerging and then suddenly increased on the fourth day (Table S3, Fig. 10). The BBE-like enzyme encoded by *Os06g0548200* is involved in plant phenylpropanoid metabolism. Quantitative results showed that the contents of shikimic acid and Phe increased with increasing submerging time (Table S3, Fig. 9).

## Discussion

In rice production in Asia, due to rising costs, labour shortages and water shortages, farmers often choose to plant directly in rain-fed and irrigation systems (Miro and Ismail 2013). In this case, the ability of rice to withstand flooding during germination is crucial. Rice seeds can germinate under hypoxic or even anaerobic conditions and extend the coleoptile to some extent (Biswas and Yamauchi 1997; Ella and Setter 1999), but growth during AG and the early seedling stage is extremely sensitive to anaerobic conditions (Yamauchi et al. 1993; Ismail et al. 2009; Angaji et al. 2010). Only tolerant genotypes have the ability to rapidly extend the coleoptile and form roots under submerged conditions in the field (Ismail et al. 2009). In contrast, the coleoptiles of sensitive genotypes grow more slowly, and the seedlings cannot develop further. It has been speculated that most rice genotypes can initiate germination but cannot complete it. Similar studies have reported the use of GWAS methods to mine functional genes for AG in rice, but few accessions have been used, and dynamic GWAS analysis examining the germination stage and evidence for targeted genes have been lacking (Hsu and Tung 2015; Zhang et al. 2017; Nghi et al. 2019). In this study, a high-density SNP marker set based on resequencing was used to perform a GWAS on the CL and CD of 209 diverse rice materials with RNA-Seq based on a differential expression profiling strategy. We identified 4 notable genes, and their expression levels were consistent with changes in the coleoptile phenotype under AG conditions. Furthermore, we used LC-MS/MS extensive targeted metabolic detection technology to verify the contents of metabolites related to the regulatory pathways associated with these four genes to support their importance in rice AG tolerance. These results may further enhance our understanding of AG in rice and help to explore the genetic mechanisms of rice AG during flooding.

The plant genome contains a large number of genes encoding BBE, and the BBE protein family is widespread in the plant kingdom and involved in plant phenylpropanoid metabolism. Phenylpropanoid metabolism is an important pathway for the synthesis of secondary metabolites in plants. All substances containing a phenylpropane skeleton are directly or indirectly produced by this pathway. These compounds have multiple functions in plants (Schadel and Walter 1981). In recent years, in the process of genome sequencing, many genes encoding BBE-like enzymes have been identified in plants and bacteria. In the model plant *Arabidopsis thaliana*, 28 BBE-like genes referred to as *AtBBE-like 1–28* (Wallner et al. 2013) were identified and found to be expressed in certain developmental stages of Arabidopsis, such as elongation and maturation, proliferation and embryonic development (Winter et al. 2007). Daniel et al. (2015) found that the two enzymes At-BBE-like 13 and At-BBE-like 15 may be involved in the mobilization and oxidation of the monolignols required for the polymerization process of the plant cell wall (such as lignification), while the BBE-like 15 enzyme family and potentially members of other related families work in concert with them to mobilize building blocks from their storage forms, participate in the cell wall formation required by Arabidopsis growth, and respond to stressors. Our dynamic GWAS correlation analysis found that *Os06g0548200* is located in a genomic region significantly correlated with CD and is highly expressed in the coleoptile under hypoxic conditions. In the early stage, we found that fructose hexaphosphate (F-6-P) is an important metabolite produced in response to AG in rice seeds. F-6-P provides energy through glycolysis and participates in the production of compounds, such as phosphoenolpyruvate (PEP), through the TCA cycle. PEP then enters phenylpropanoid metabolism through the synthesis of shikimic acid and phenylalanine (Phe) (Fig. 9). The results of our qualitative and quantitative metabolite analysis of YZX seeds during AG showed that the contents of shikimic acid and Phe increased with increasing submergence time (Table S3, Fig. 10), consistent with the expression level of *Os06g0548200*.

The *OsGA2ox8* (*Os05g0560900*) gene is located in a region that was significantly associated with CD at both AN3d and AN4d. It encodes gibberellin 2-beta-dioxygenase, which controls participation in gibberellin (GA) catabolism. GAs are a class of endogenous plant hormones that regulate growth and development throughout the life cycle of higher plants (Hooley 1994), including seed germination, hypocotyls and stem elongation, leaf extension, epidermal trichome development, flowering time, floral organ development, and fruit ripening (Yamaguchi et al. 1998; Itoh et al. 1999). For the normal growth and development of plants, it is very important to produce and maintain the optimal level of bioactivity GAs (GA1, GA3, GA4 and GA7) (Lo et al. 2017; Hedden and Phillips 2000). The concentration of GA is regulated through both synthesis and inactivation. The main GA inactivation pathway is through GA 2-oxidation, which regulates GA concentration (Rieu et al. 2008). The main catabolic pathway of GA is 2 β - hydroxylation catalyzed by GA2ox. The common C_19_-GA2oxs class can reduce the level of bioactive GA. It can reduce the C-2 hydroxyl of active C_19_-GA precursors (GA_9_ and GA_20_) and C_20_-GA precursors (GA_12_,GA_53_) (Lo et al. 2008; Sakamoto et al. 2003; Yamaguchi 2008), synthesis of bioactive GA_1_, GA_3_, GA_4_ and GA_7_. In Arabidopsis, *ga2ox7* and *ga2ox8* mutants had higher germination rates and longer coleoptiles under the action of GA biosynthetic inhibitor Pyrimidinol (Schomburg et al. 2003). Lo et al. (2008) found that moderate down-regulation of *GA2ox5* and *GA2ox9* in DAI 2 was associated with rapid seed germination. We found that *GA2ox8* expression was up-regulated and GA_9_, GA_15_ and GA_20_ contents were decreased under hypoxia stress in rice seeds, indicating that C_20_-GA2ox may play a coordinating role in regulating GA content required for rice seed anoxic germination. However, it is not clear how GA2ox is differentially regulated during seed anoxic germination. We speculate that *OsGA2ox8* gene may be involved in GA catabolism and inactivate the direct precursors of three kinds of bioactive GA, so as to regulate the endogenous level of bioactive GAs (such as GA_1_, GA_4_) and the coleoptile elongation of rice seeds. Due to the complex feedback regulatory network and the spatiotemporal expression of GA2oxs and other enzymes involved in GA biosynthesis and catabolism, as well as the interaction between GA metabolism and reaction pathways (Olszewski et al. 2002; Yamauchi et al. 2007), so we are now trying to explain the regulation mechanism of *GA2ox8* expression through broader biochemical and genetic interference studies to better understand its exact function in rice hypoxia germination.

The plant hormone ABA plays central roles in seed maturation, germination and adaptation to abiotic environmental stress (Leung and Giraudat 1998). In rice, *OsVP1*(*Os01g0911700*) is a direct homologous gene in maize *ABI3*, encoding a B3 domain-containing transcription factor necessary for seed response to ABA (Hattori et al. 1994; Hobo et al. 1999). *OsABI5* is a homolog of *ABI5* in *Arabidopsis thaliana* (Nijhawan et al. 2008), Zou et al. (2007) found that two alternative splicing mutants of the bZIP type transcription factor *OsABI5*, *OsABI5–1* and *OsABI5–2*, may have overlapping and different functions in the regulation of downstream target genes, and regulate ABA signal as a transcription factor to express with *OsVP1*. In our dynamic GWAS analysis, *Os01g0911700* was found in an area significantly correlated with CD at AN2d. The expression was up-regulated in hypoxia and down regulated in aerobic environment (Fig. 7). ABA content fluctuated at a low level all the time, suggesting the hypoxic stress caused by flooding conditions, which caused *OsVP1* to fine tune the ABA signaling pathway (Fig. 9) and promoted seed germination. In addition, Mohanty et al. (2012) through the enrichment analysis of cis elements of up-regulated and down-regulated genes responding to hypoxia, it was found that specific ABA responsive cis elements, MYB-box and ethylene responsive cis element were highly enriched in the up-regulated genes. They interacted with specific transcription factors bZIP, MYB and ERF, resulting in hypoxia regulation and acting as the main regulator of rice germination under hypoxia conditions, common to adjust coleoptile elongation. It is well known that ABA involved in regulation of seed dormancy and germination inhibition, this seemingly contradictory and our study, but the low oxygen adversity caused by abiotic stress on seed germination in the process of different hormone regulation may exist between collaborative crosstalk effect, this may be related to hormonal regulation in the process of normal germination differences, this is very interesting and worth our further research.

*OsDi19–1* (*Os05g0562200*) encodes the dehydration-induced Di19 protein, a member of a small family of plant transcription factors, and is reported to be involved in abiotic stress (Liu et al. 2013; Wang et al. 2016). *OsDi19–4*, a member of the rice Di19 protein family, has been reported to act as a transcription factor. Through the *OsCDPK14* – *OsDi19–4* – *OsASPG1*/*OsNAC18* regulatory pathways, it not only indirectly regulates a large number of plant stress and ABA response genes but also, by interacting with their promoters, directly regulates certain ABA-responsive genes (such as *OsASPG1* and *OsNAC18*), which further modulate ABA-related phenotypes (Wang et al. 2016). In our dynamic GWAS analysis, *OsDi19–1* was detected at both AN3d and AN4d, and its expression level increased with time under anaerobic conditions and when plants were transferred to anaerobic from aerobic conditions. Under aerobic conditions, its expression level decreased; thus, it specifically responded to a hypoxic environment. We quantitatively analysed ABA in YZX seeds at different time points under anaerobic germination conditions and found that the content of ABA gradually decreased in the first 3 days after flooding but suddenly increased on the 4th day. Under timed oxygen-deficiency conditions, *OsDi19–1* directly or indirectly regulates ABA-related response genes, tweaking ABA content, which in turn regulates the coleoptile phenotype (Table S3, Fig. 9).

Three of the 4 candidate genes identified by our dynamic GWAS association analysis results (*Os05g0560900*, *Os01g0911700*, *Os05g0562200*) were related to the plant hormones ABA and GA, which indicated that ABA and GA play important roles in coleoptile elongation during AG in rice seeds. During the anoxic germination of rice seeds, the main energy supply was glycolysis and fermentation metabolism. During this period, cross-talk between different hormone-mediated pathways may occur to maintain ATP level and regulate sugar consumption (Mohanty et al. 2012). Quantitative analysis of GA and ABA in submerged YZX seeds showed that GA content decreased to a plateau over time, and ABA content decreased first and then increased (Table S3, Fig. 10). After 0–3 days of submergence, the GA content was 2–3 times that of ABA; after 4 days of flooding, the GA content reached a minimum, and the ABA content increased to 185 times that of GA. These results indicate that although GA and ABA both play roles in coleoptile elongation under submerged conditions, their regulatory responses have temporal and spatial differences. How these three candidate genes participate in regulating the GA and ABA signalling regulatory pathways will be further explored in future work.

## Conclusion

In this study, high-density SNP markers based on resequencing were used to perform dynamic GWAS on the coleoptile phenotypes of 209 natural rice populations, and four reliable candidate genes were identified. These genes were involved in GA and ABA signalling and cell wall metabolic processes, showed differential expression under AG conditions, and promoted coleoptile elongation, allowing seedlings to escape immersion by extending the coleoptile out of the anaerobic environment to obtain oxygen and support rapid germination.

## Materials and Methods

### Plant Material and Growth Conditions

The mapping population consisted of 209 rice accessions (Yang et al. 2019a, 2019b). The majority came from Southern China, whereas others were from Japan, the Philippines, Vietnam, Indonesia, Pakistan, India, the United States and other countries (Table S1). Seeds of the population were planted in a paddy field at South China Agricultural University, Guangzhou (23°16′ N, 113°8′ E), during the late cropping season (July–November) in 2018. The spaces between rows and between plants were 20 and 20 cm, respectively. Thirty-six plants of each accession were grown in 6 rows with 6 plants per row. Considering that seed maturity affects anaerobic germination (AG), six individual plants in the middle of each block were harvested independently on the 40th day after heading in the late cropping season. Crop management and disease and insect pest control were performed as locally recommended. The harvested seeds were dried in a heated air dryer at 42 °C for 5 days and then stored at − 20 °C (Yang et al. 2019a, 2019b).

### Phenotyping

From the six independently harvested plants, 30 healthy, fully mature seeds were selected for each accession. The seeds of each experimental cultivar were placed in an oven at 50 °C for 5 days to break dormancy, surface-sterilized with 0.2% HgCl_2_ for 5 min and then washed thrice with sterile distilled water. Five seeds were placed in one sterile centrifuge tube (50 mL), which was filled with distilled water to create anaerobic conditions. Germination took place at 30 °C in a growth chamber in the dark. After 2, 3, and 4 days, a WinRHIZO (Regent Instruments Inc., Québec, Canada) root image analysis system was used to measure the coleoptile length (CL), coleoptile surface area (CSA), coleoptile volume (CV), and coleoptile diameter (CD). Three independent, replicated experiments were performed for each rice accession. All treatments were performed in parallel. Statistical analysis was performed with SPSS (Statistical Analysis System, version 23.0) and Microsoft Excel.

### Whole-Genome Resequencing and Variation Detection

The genomes of all 209 accessions were sequenced on the Illumina HiSeq 2500 Sequencing Systems Platform (Illumina Inc. USA), with an average coverage of approximately 12×. Raw reads were processed to obtain an average quality score (QS) per read ≤30 by trimming the 3′ ends of the reads using SICKLE (https://github.com/najoshi/sickle). High-quality reads were aligned against the rice reference genome (IRGSP 1.0) (Kawahara et al. 2013) for genotype calling using Burrows - Wheeler Aligner (BWA) (version 0.7.5a).

Single nucleotide polymorphisms (SNPs) were mainly detected by the GATK (McKenna et al. 2010) software kit. According to the positioning results of clean reads in the reference genome, Picard (http://sourceforge.net/projects/picard/) was used to filter the redundant reads (mark duplicates) to ensure the accuracy of the detection results. Finally, low-quality markers, deletion markers < 25% and minor allele frequency (MAF) > 5% were filtered, and the final set of mutation loci was obtained.

### Population Structure Analysis and Genome-Wide Association Mapping

Principal component analysis (PCA), construction of a neighbour-joining (NJ) tree, determination of linkage disequilibrium (LD) decay level and kinship analysis among the landraces were performed based on SNPs. The population structure of the 209 varieties was estimated with PCA by using the software EIGENSTRAT (Tamura et al. 2007). PHYLIP version 3.695 software (http://evolution.genetics.washington.edu/phylip/getme-new1.html) was used to construct the NJ tree on the basis of similarity measures, and MEGA V6.0 was used to observe the NJ tree (Purcell et al. 2007). A Q matrix was obtained from the membership probability of each variety using ADMIXTURE version 1.22 software (Hardy and Vekemans 2002). The Q matrix was used for further association mapping. The Loiselle algorithm was chosen to construct a kinship matrix (K) with the software SPAGeDi (Kang et al. 2010). Moreover, all negative kinship values were set to zero.

Based on the developed high-density SNP molecular marker data, this study used TASSEL 4.0 (Bradbury et al. 2007), FaST-LMM (Lippert et al. 2011), and EMMAX (Yang et al. 2014) for correlation analysis. Association analysis was implemented under a mixed linear model (MLM), which could be described as y = *X*α + *S*β + *K*μ + e. *X* and y represented the genotype and phenotype, α represented the SNP effects, β represented the effects of population structure, μ was the vector of kinship background effects, e corresponded to residual effects, *S* was the PCA matrix relating y to β, and *K* represented the related kinship matrix (Wen et al. 2014; Zhang et al. 2017;Yang et al. 2019a, 2019b).

The whole set of 2,123,725 SNPs was used in association mapping with a MAF criterion of 5%. A genome-wide threshold of -log(*P*) = 7.3271 was calculated from the formula “-log10(0.1/effective number of SNPs)” (Wu et al. 2015).

### LD Structure and Haplotype Analysis

Using the software plink2 (Purcell et al. 2007), on the same chromosome, the LD between pairs of SNPs within a certain distance range (1000 kb) was calculated. When the LD value of the two markers dropped to half of the maximum value (*R*^2^ = 0.3), the interval was considered a candidate region where potential causal variants might reside. Gramene Mart (http://www.gramene.org/) was used to search for candidate genes in the target region (Hsu and Tung 2015).

Haplotyping of the identified candidate genes was carried out using DnaSP v5 (Librado and Rozas 2009). Candidate gene sequence analysis was based on the rice reference genome (IRGSP 1.0) to capture the nucleotide variation in the whole region of each target gene.

### RNA-Seq and Data Analysis of Gene Expression Profiles

An anoxia-tolerant indica rice variety (YZX) was selected from 209 natural populations and cultured for 4 days at 30 °C under low oxygen conditions (5 seeds with hulls were put into a 50 ml sterile centrifuge tube filled with sterile water); at the same time, a conversion treatment was carried out (after 3 days of cultivation under anaerobic conditions, they were transferred to normoxic conditions for 1 day). Each treatment was repeated 3 times, and 3-g samples of each replicate were collected every day, wrapped in foil, and frozen in liquid nitrogen at − 80 °C for storage. For total RNA extraction, each sample was homogenized in liquid nitrogen with a mortar and pestle, and then, the RNA was purified in accordance with the manufacturer’s instructions using the Plant Total RNA Purification Kit (Comwin Biotechnology Company).

After the total RNA was extracted from the sample (seeds + coleoptiles), the mRNA was enriched with magnetic beads with oligo (DT), and fragmentation buffer was added to the mRNA to produce short fragments. Then, first-strand cDNA was synthesized with six-base random primers, and the second strand was synthesized with buffer, dNTPs, RNase H and DNA polymerase I. After purification with the QIAquick PCR kit and elution with EB buffer, the ends were repaired, A bases were added, and then, the target fragments were recovered by agarose gel electrophoresis. PCR amplification was used to complete the preparation of the whole cDNA library. The cDNA library was sequenced on an Illumina sequencing platform (Illumina HiSeq™ 2500) by Gene Denovo Co. (Guangzhou, China). The reads obtained from the sequencing machines included raw reads containing adapters or low-quality bases, which would have affected the subsequent assembly and analysis. Thus, to obtain high-quality clean reads, the reads were further filtered according to the following rules: 1) removing reads containing adapters; 2) removing reads containing more than 10% unknown nucleotides (N); and 3) removing low-quality reads containing more than 50% low-quality (Q-value≤20) bases. All the reads that passed the filter specifications were mapped to the reference genome IRGSP-1.0, all transcripts were obtained, and their abundance was estimated by genome-guided transcription assembly using the Cufflinks package (Trapnell et al. 2012).

Differential analysis of each transcript and gene was performed using edger (Robinson et al. 2010). The error detection rate (FDR) was used to determine the threshold value of the *P*-value for multiple experiments. FDR ≤ 0.05 and absolute value of log_2_ FC ≥ 1 were used to determine the significance of gene expression differences.

### Validation of Gene Expression by qRT-PCR

For qRT-PCR, total RNA was extracted from rice coleoptiles after 2, 3, and 4 days of germination using a MiniBEST Plant RNA Extraction kit (Takara Bio, Inc., Japan). According to the method of Guo et al. (2019), the candidate genes were analysed by qRT-PCR, and NCBI Primer BLAST (http://www.ncbi.nlm.nih.gov/tools/primer-blast/) was used to design gene-specific primers. The primer sequences of the 4 candidate genes are shown in Table S5.

### Detection of Metabolites in Seeds under Anaerobic Conditions by LC-MS/MS

To understand the metabolic process of rice seed germination under anaerobic conditions, YZX was selected for metabolite detection among the 209 accessions, and LC-MS/MS was used to detect metabolites. The test seeds were placed in an oven at 50 °C for 5 days to break dormancy, surface sterilized with 0.2% HgCl_2_ for 5 min and washed with sterile distilled water 3 times. Groups of 5 seeds were placed in sterile centrifuge tubes (50 ml), which were filled with distilled water to achieve anaerobic conditions and germinated in a constant-temperature incubator at 30 °C in darkness. After 2, 3 and 4 days of AG, three biological repeats were taken from each treatment, and dry seeds were used as a control. The methods of Chen et al. (2013) were used for sample preparation, extraction and qualitative and quantitative determination of metabolites. The metabolite database used included the MWDB database established by Metware company, the metabolite information public database (MassBank (http://www.massbank.jp), KNApSAcK (http://kanaya.naist.jp/KNApSAcK), Human Metabolome Database (HMDB; http://www.hmdb.ca), MoTo DB (http://www.ab.wur.nl/moto), and METLIN (http://metlin.scripps.edu/index.php)).

## Supplementary Information

**Additional file 1: Fig. S1.** Variation detection and annotation of SNP. a, Mutation detection of SNP in 209 rice accessions. b,SNP annotation of 209 rice accessions

**Additional file 2: Fig. S2.** The linkage disequilibrium (LD) decay of marker- pairs over all chromosomes for the population

**Additional file 3: Table S1.** Source of 209 rice accessions materials. SD means standard deviation;SE stands for standard error; CV is the coefficient of variation. Sheet 1 is the coleoptile phenotype data after 2 days of submersion; Sheet 2 is the coleoptile phenotype data after 3 days of submersion; Sheet 3 is the coleoptile phenotype data after 4 days of submersion

**Additional file 4: Table S2.** Phenotype of 209 rice accessions during germination under submerging

**Additional file 5: Table S3.** Detected metabolites. Note: Mix 1–10 are quality control (QC) samples

**Additional file 6: Table S4.** Contents of seven related metabolites regulated by candidate genes

**Additional file 7: Table S5.** Haplotype and phenotype association analysis of candidate genes in High AGs and Low AGs. Note: unit, cm

**Additional file 8: Table S6.** qRT-PCR primer sequences of four candidate genes

**Additional file 9: Table S7.** qRT-PCR expression profile of four most promising candidate genes in different oxygen environments

**Additional file 10: Table S8.** Expression levels of 106 candidate genes in Nipponbare under anoxia and aerobic treatments (transcriptome data from Lasanthi-Kudahettige et al. (2007))

**Additional file 11: Table S9.** The significant SNPs for coleoptiles length (CL) and diameter (CD) under anaerobic germination (AG) using genome-wide association. Note: a The SNP positions were based on the annotation data on Os-Nipponbare-Reference-IRGSP-1.0 (RAP-DB, http://rapdb.dna.affrc.go.jp/); b Position of the SNP showing the most significant association for AG

**Additional file 12: Fig. S3.** MRM multimodal map for metabolite detection. The panel a is the detection in the positive ion mode; the panel b is the detection in negative ion mode

## Data Availability

All the RNA-seq data generated in this research was deposited in the Sequence Read Archive database (www.ncbi.nlm.nih.gov/sra) at NCBI (National Center for Biotechnology Information) under accession number: SRP282222. The data sets supporting the results of this study are included in the manuscript. Rice seeds are available from the National Engineering Research Center of Plant Space Breeding, PR China.

## Abbreviations

AG: Anaerobic germination
CL: Coleoptile length
CSA: Coleoptile surface area
CD: Coleoptile diameter
CV: Coleoptile volume
DSR: Direct-seeded rice
GWAS: Genome-wide sequencing analysis
LC-MS/MS: Liquid chromatography tandem mass spectrometry
