# Supplementary figures and images for: Dynamic genome-wide association analysis and identification of candidate genes involved in anaerobic germination tolerance in rice
Source: Rice (N Y). 2021 Jan 6;14:1. doi: 10.1186/s12284-020-00444-x (PMC7788155; doi:10.1186/s12284-020-00444-x)

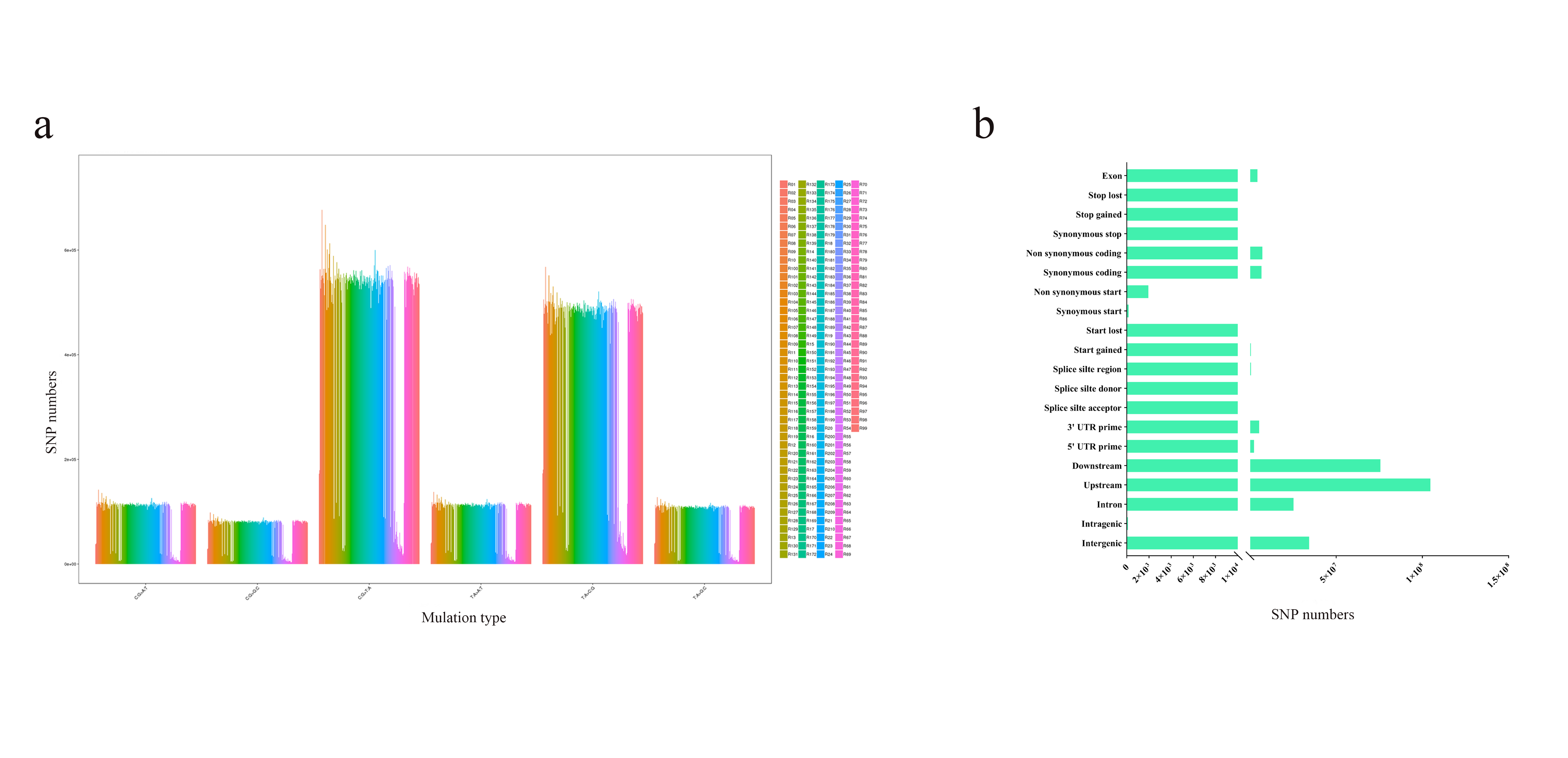

Supplement: Supplementary file 1 — Additional file 1: Fig. S1. Variation detection and annotation of SNP. a, Mutation detection of SNP in 209 rice accessions. b,SNP annotation of 209 rice accessions [file 12284_2020_444_MOESM1_ESM.tif]

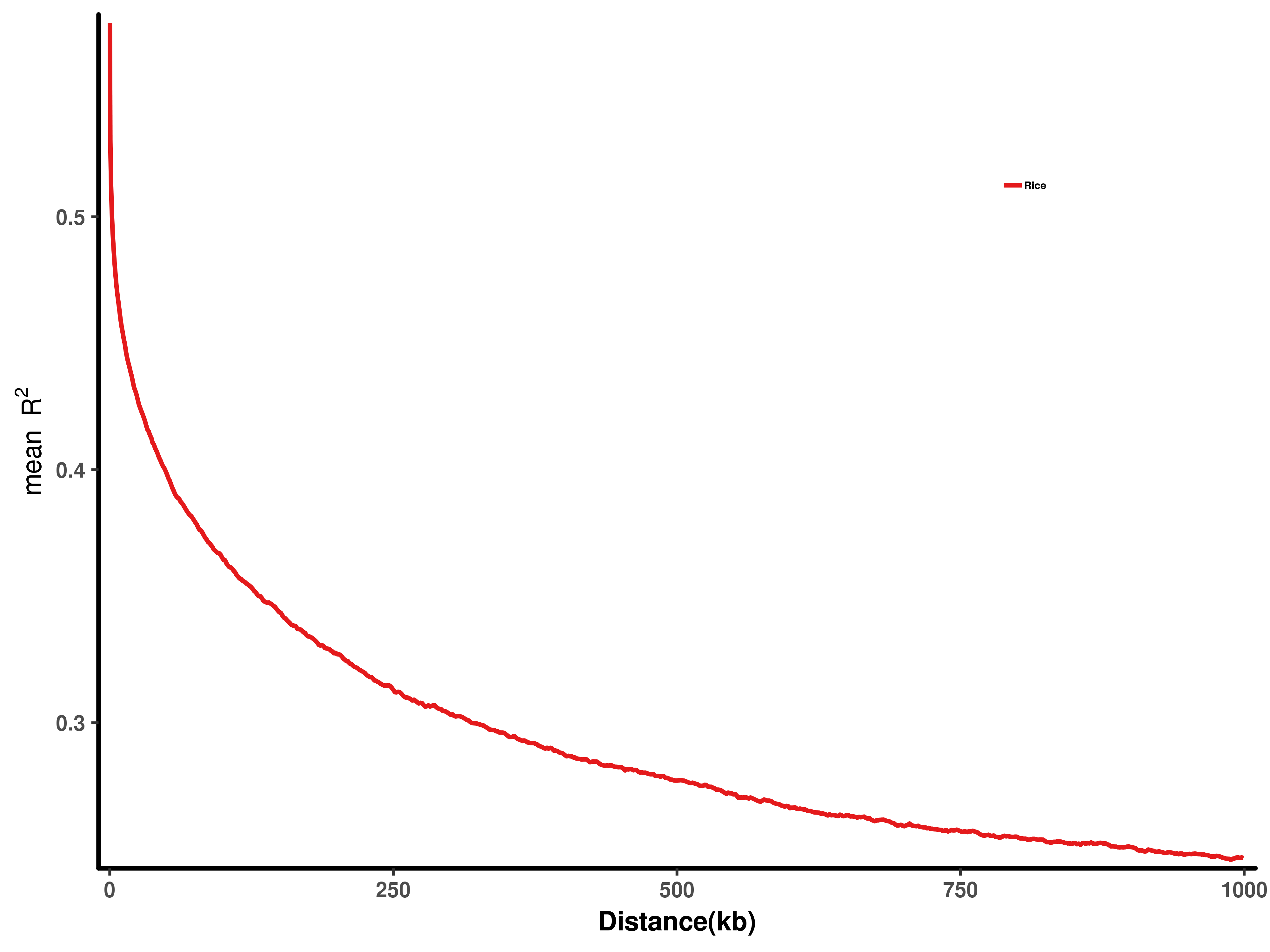

Supplement: Supplementary file 2 — Additional file 2: Fig. S2. The linkage disequilibrium (LD) decay of marker- pairs over all chromosomes for the population [file 12284_2020_444_MOESM2_ESM.tif]

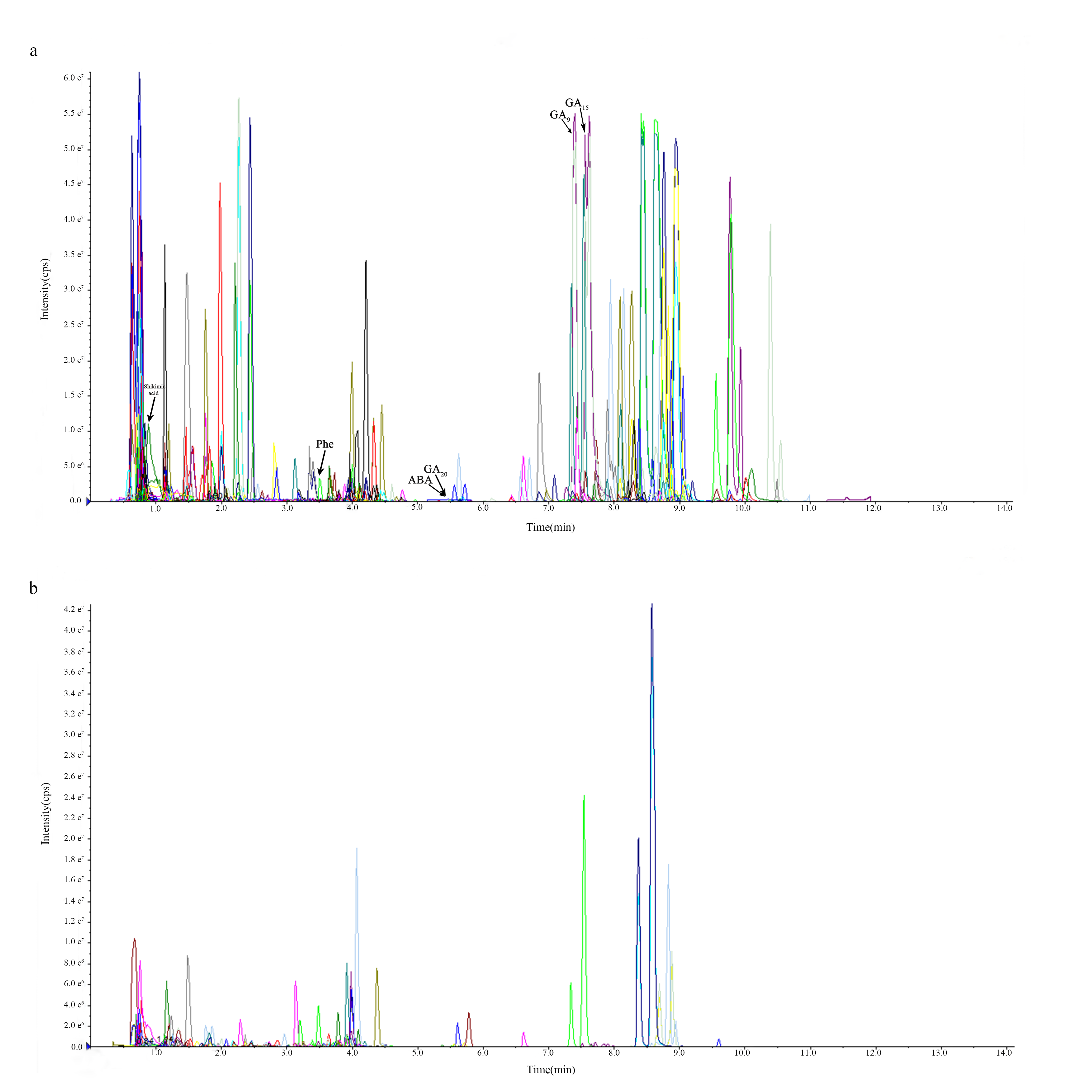

Supplement: Supplementary file 12 — Additional file 12: Fig. S3. MRM multimodal map for metabolite detection. The panel a is the detection in the positive ion mode; the panel b is the detection in negative ion mode [file 12284_2020_444_MOESM12_ESM.tif]
